# Involvement of circulating CEA in liver metastases from colorectal cancers re-examined in a new experimental model

**DOI:** 10.1038/sj.bjc.6690531

**Published:** 1999-07

**Authors:** A Leconte, V Garambois, M Ychou, B Robert, D Pourquier, A Terskikh, J P Mach, A Pèlegrin

**Affiliations:** Centre de Recherche en Cancérologie, CRLC Val d'Aurelle – Paul Lamarque Cancer Centre, Parc Euromédecine, Montpellier, Cedex 5, F-34298, France; Institut de Biochimie, Université de Lausanne, Suisse

**Keywords:** serum CEA, colon cancer, liver metastases, experimental model

## Abstract

Both experimental and clinical data show evidence of a correlation between elevated blood levels of carcinoembryonic antigen (CEA) and the development of liver metastases from colorectal carcinomas. However, a cause–effect relationship between these two observations has not been demonstrated. For this reason, we developed a new experimental model to evaluate the possible role of circulating CEA in the facilitation of liver metastases. A CEA-negative subclone from the human colon carcinoma cell line CO115 was transfected either with CEA-cDNA truncated at its 3′ end by the deletion of 78 base pairs leading to the synthesis of a secreted form of CEA or with a full-length CEA-cDNA leading to the synthesis of the entire CEA molecule linked to the cell surface by a GPI anchor. Transfectants were selected either for their high CEA secretion (clone CO115-2C2 secreting up to 13 μg CEA per 10^6^ cells within 72 h) or for their high CEA membrane expression (clone CO115-5F12 expressing up to 1 × 10^6^ CEA molecules per cell). When grafted subcutaneously, CO115-2C2 cells gave rise to circulating CEA levels that were directly related to the tumour volume (from 100 to 1000 ng ml^−1^ for tumours ranging from 100 to 1000 mm^3^), whereas no circulating CEA was detectable in CO115 and CO115-5F12 tumour-bearing mice. Three series of nude mice bearing a subcutaneous xenograft from either clone CO115-2C2 or the CO115-5F12 transfectant, or an untransfected CO115 xenograft, were further challenged for induction of experimental liver metastases by intrasplenic injection of three different CEA-expressing human colorectal carcinoma cell lines (LoVo, LS174T or CO112). The number and size of the liver metastases were shown to be independent of the circulating CEA levels induced by the subcutaneous CEA secreting clone (CO115-2C2), but they were directly related to the metastatic properties of the intrasplenically injected tumour cells. © 1999 Cancer Research Campaign


*British Journal of Cancer* (1999) **80**(9), 1373–1379
© 1999 Cancer Research Campaign
Article no. bjoc.1999.0531

### Involvement of circulating CEA in liver metastases from colorectal cancers re-examined in a new experimental model

**A Leconte[1], V Garambois[1], M Ychou[1], B Robert[1], D Pourquier[1], A Terskikh[2], JP Mach[2] and A Pèlegrin[1]**

[1]Centre de Recherche en Cancérologie, CRLC Val d'Aurelle – Paul Lamarque Cancer Centre, Parc Euromédecine, F-34298 Montpellier, Cedex 5, France;
[2]Institut de Biochimie, Université de Lausanne, Suisse

**Summary** Both experimental and clinical data show evidence of a correlation between elevated blood levels of carcinoembryonic antigen (CEA) and the development of liver metastases from colorectal carcinomas. However, a cause–effect relationship between these two observations has not been demonstrated. For this reason, we developed a new experimental model to evaluate the possible role of circulating CEA in the facilitation of liver metastases. A CEA-negative subclone from the human colon carcinoma cell line CO115 was transfected either with CEA-cDNA truncated at its 3′ end by the deletion of 78 base pairs leading to the synthesis of a secreted form of CEA or with a full-length CEA-cDNA leading to the synthesis of the entire CEA molecule linked to the cell surface by a GPI anchor. Transfectants were selected either for their high CEA secretion (clone CO115-2C2 secreting up to 13 µg CEA per $10^6$ cells within 72 h) or for their high CEA membrane expression (clone CO115-5F12 expressing up to $1 \times 10^6$ CEA molecules per cell). When grafted subcutaneously, CO115-2C2 cells gave rise to circulating CEA levels that were directly related to the tumour volume (from 100 to 1000 ng $ml^{-1}$ for tumours ranging from 100 to 1000 $mm^3$), whereas no circulating CEA was detectable in CO115 and CO115-5F12 tumour-bearing mice. Three series of nude mice bearing a subcutaneous xenograft from either clone CO115-2C2 or the CO115-5F12 transfectant, or an untransfected CO115 xenograft, were further challenged for induction of experimental liver metastases by intrasplenic injection of three different CEA-expressing human colorectal carcinoma cell lines (LoVo, LS174T or CO112). The number and size of the liver metastases were shown to be independent of the circulating CEA levels induced by the subcutaneous CEA secreting clone (CO115-2C2), but they were directly related to the metastatic properties of the intrasplenically injected tumour cells.

**Keywords:** serum CEA; colon cancer; liver metastases; experimental model

In patients with colorectal carcinoma, high levels of circulating carcinoembryonic antigen (CEA) are associated with poor prognosis. After surgical removal of a primary tumour, increasing circulating CEA levels often precede the development of metastases, especially liver metastases (Lucha et al, 1997). Thus, there is a clear relationship between circulating CEA levels and metastases, but it is impossible to demonstrate in humans a connection between cause and effect.

Several authors have demonstrated that CEA can be considered as an adhesion molecule (Benchimol et al, 1989; Oikawa et al, 1989; Pignatelli et al, 1990). The question addressed in the present study was to determine whether these adhesive properties facilitate the development of liver metastases from colorectal cancer. Up to now, the involvement of CEA in the development of liver metastases from colorectal cancers has been addressed in two different ways: (i) comparison of the metastatic potential of different human colon carcinoma cell lines or CEA-transfected cells in association with CEA membrane expression (Tibbetts et al, 1993; Thomas et al, 1995; Landuzzi et al, 1996); (ii) analysis of the effect of circulating CEA on the development of liver metastases from different human colon carcinoma cells (Hostetter et al, 1990; Jessup et al, 1993*a*). This second approach is particularly interesting since one can imagine different strategies for modifying circulating CEA levels such as its capture with anti-CEA monoclonal antibodies (mAb). If there is a cause–effect relationship between circulating CEA levels and the development of liver metastases from colorectal cancer, the elimination of CEA from the blood stream in patients would be expected to reduce the possibility of their developing such metastases.

*Received 28 August 1998*
*Revised 4 January 1999*
*Accepted 12 January 1999*

*Correspondence to:* A Pèlegrin

To test this hypothesis, instead of injecting CEA into the bloodstream of tumour-bearing nude mice, we propose a new animal model in which a CEA-secreting subcutaneous (s.c.) tumour gives rise to different circulating CEA levels as a function of the tumour volume. Using this model, we induced liver metastases from three different colon carcinoma cell lines expressing high levels of membrane CEA (LS174T, LoVo and CO112). We demonstrated that the number and size of the liver metastases depended on the cell line and the eventual resection of the s.c. tumour but were not related to the circulating CEA levels.

### MATERIALS AND METHODS

### Cell lines

The LS174T (ATCC CL188) and the LoVo (ATCC CCL229) cell lines were purchased from the American Type Culture Collection. The CO112 cell line was established by Mach et al (1974). These three human colon carcinoma cell lines were used as positive controls in the screening and characterization of the transfectants and for metastatic potential assay. The CEA-negative subclone from

**Table 1** Experimental liver metastasis after intrasplenic injection of LoVo, LS174T or CO112 cells in nude mice bearing CO115, CO115-5F12 or CO115-2C2 tumours

| Intrasplenic injection | Subcutaneous tumour | Subcutaneous tumour resection | Sacrifice | Mice (evaluable/ included) | Liver metastasis grade[a] | | | | | Circulating CEA (ng/ml) | | |
|---|---|---|---|---|---|---|---|---|---|---|---|---|
| | | | | | 0 | I | II | III | IV | D-1[b] | D+7 | D+15 |
| LoVo | CO115–2C2 | – | D+29 | 12/13 | 9 | | 3 | | | 46–400 (173)[c] | – | – |
| | – | – | D+29 | 11/11 | 9 | 2 | | | | < 5 | – | – |
| LoVo | CO115–5F12 | D+7 | D+42 | 8/8 | 5 | 1 | 2 | | | < 5 | < 5 | < 5 |
| | CO115–2C2 | D+7 | D+42 | 12/12 | 7 | 3 | 2 | | | 24–818 (238) | 252–1970 (842) | < 5 |
| LoVo | CO115 | D+7 | D+42 | 6/6 | 4 | | 2 | | | < 5 | < 5 | < 5 |
| | CO115–5F12 | D+7 | D+42 | 6/6 | 5 | 1 | | | | < 5 | < 5 | < 5 |
| | CO115–2C2 | D+7 | D+42 | 7/8 | 4 | 1 | 2 | | | 23–192 (119) | 157–600 (364) | < 5 |
| LS174T | CO115 | – | D+11 | 6/6 | 3 | | 3 | | | < 5 | – | – |
| | CO115–5F12 | – | D+11 | 6/6 | 2 | | 4 | | | < 5 | – | – |
| | CO115–2C2 | – | D+11 | 6/6 | 1 | | 3 | 2 | | 22–266 (128) | – | – |
| | – | – | D+11 | 2/2 | | | 2 | | | < 5 | – | – |
| LS174T | CO115 | D+7 | D+27 | 3/6 | 1 | 1 | 1 | | | < 5 | < 5 | < 5 |
| | CO115–5F12 | D+7 | D+27 | 5/6 | | 1 | 1 | 1 | 2 | < 5 | < 5 | < 5 |
| | CO115–2C2 | D+7 | D+27 | 7/8 | 1 | | 2 | 2 | 2 | 150–533 (298) | 147–957 (524) | < 5 |
| CO112 | CO115 | D+7 | D+27 | 5/6 | | 1 | 1 | 1 | 2 | < 5 | < 5 | < 5 |
| | CO115–5F12 | D+7 | D+27 | 6/6 | | 2 | | 1 | 3 | < 5 | < 5 | < 5 |
| | CO115–2C2 | D+7 | D+27 | 6/8 | | 1 | 1 | 2 | 2 | 113–500 (404) | 141–977 (439) | < 5 |

[a]Tumour burden was assessed according to the classification of Giavazzi et al (1986): 0, no tumour deposit, tumour-free; I, histological evidence of tumour growth or one isolated metastasis; II, fever than ten tumour foci, < 1–2 mm in diameter; III, 10–100 tumour foci, 3–5 mm in diameter; IV, more than 100 tumour foci, > 5 mm in diameter. Each value represents the number of mice for each metastatic grade. [b]All the dates are expressed in days (D), D0 being the day of intrasplenic injection of LoVo, LS174T or CO112 cells. [c]Range. Average value is indicated in parenthesis.

human colon carcinoma cell line CO115 was established in one of our laboratories (Mach et al, 1974; Carrel et al, 1976). The cells were transfected with truncated CEA-cDNA as described in a previous study where the isolation of the CO115–2C2 clone is reported. In culture, CO115–2C2 secretes up to 13 µg fully immunoreactive CEA per $10^6$ cells within 72 h (Terskikh et al, 1993).

### Full-length CEA-cDNA transfection

CO115 cells were transfected with pRc/CMV/CEA-cDNA (Pèlegrin et al, 1992) using a mammalian transfection kit (Stratagene, La Jolla, CA, USA). Three µg of DNA were precipitated with calcium phosphate according to the manufacturer's instructions and incubated for 16 h with about $3 \times 10^6$ non-confluent adherent carcinoma cells. Fresh culture medium was then added. After a further 24-h incubation, the cells were harvested and divided in two 75 $cm^2$ culture flasks and grown for 24 h before adding the Neomycin analogue G418 (Gibco, Paisley, UK) at a concentration of 200 µg $ml^{-1}$. This concentration of G418 was shown to be three times the minimal lethal dose for non-transfected CO115 cells. Cells resistant to G418 were expanded and tested for CEA expression by flow cytometry using a fluorescence-activated cell sorter (FACS) (Becton-Dickinson, Mountain View, CA, USA). CEA-positive cells were sorted and cloned by limiting dilution.

### FACS analysis

Cells were incubated for 15 min at 37°C with trypsin (2.5 mg $ml^{-1}$), washed once in RPMI medium 10% fetal calf serum (FCS) and then washed twice in phosphate-buffered saline (PBS) containing 1 mg $ml^{-1}$ bovine serum albumin (BSA). This buffer was used for all the washing steps, except for the last one in which BSA was omitted. For CEA-cDNA transfectant screening, about $5 \times 10^5$ cells were incubated for 1 h with a pool of four anti-CEA mAb (35A7, CE25, B93, B17) labelled with fluoroscein isothiocyanate (FITC Isomer I, Sigma, St Louis, MO, USA) and then washed twice before FACS analysis. For CEA epitopes analysis, five mAb directed against the five CEA epitopes were used as primary antibody followed by a fluorosceine-labelled sheep anti-mouse $F(ab')_2$ as secondary antibody (Silenus, Victoria, Australia). Fluoroscein-labelled or unlabelled normal mouse $IgG_1$ was used for background measurement. The samples were analysed on a FACScanII (Becton-Dickinson). Each figure represents data obtained from the analysis of 10 000 cells.

The GPI anchorage of CEA was analysed by phosphatidylinositol-specific phospholipase C (PI-PLC) treatment of the cells before FACS analysis. Five to $10 \times 10^6$ tumour cells were treated with 0.5–1.0 unit PI-PLC (Boehringer Mannheim, Germany) for 1 h at 37°C in PBS containing 1 mg $ml^{-1}$ BSA.

### Immunocytochemical analysis

Cells were centrifuged on SuperFrost Plus microscope slides and fixed in methanol:acetone (1:1) for 10 min at –20°C. Endogenous peroxidase activity was inhibited by incubating sections in 0.3% hydrogen peroxide for 5 min. The anti-CEA chimeric mouse-human mAb X4 (Hardman et al, 1989) was used as primary antibody (10 µg $ml^{-1}$ for 30 min) followed by incubation for 30 min with horseradish peroxidase-conjugated anti-human IgG (Dako, P214; diluted 1:50). The staining reaction was performed using AEC as substrate. Slides were counterstained with haematoxylin.

 *© 1999 Cancer Research Campaign*

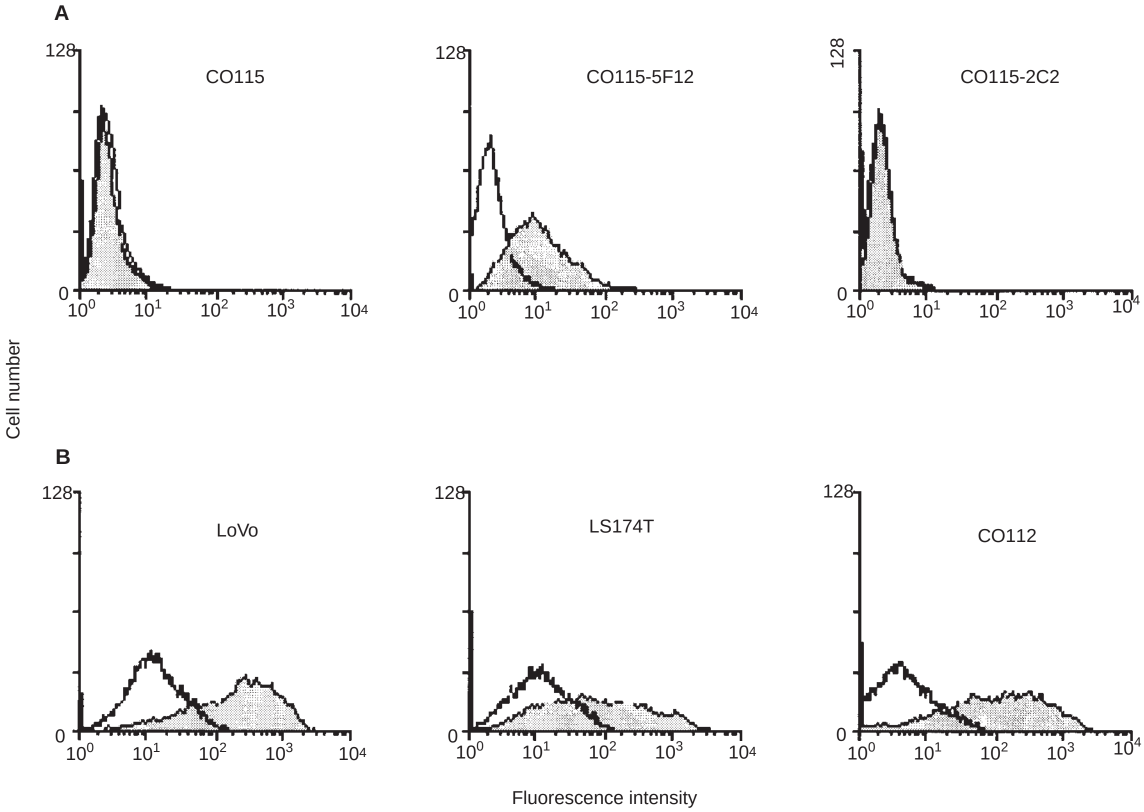

**Figure 1** (**A**) Flow cytometry analysis of full-length and truncated CEA-cDNA transfected clones and the parental cell line CO115. mAb 35A7 specific for the CEA Gold 2 epitope (grey shading) and an irrelevant IgG (no shading) were used as primary antibody. CO115: parental CEA-negative human colon carcinoma cell line; CO115-5F12: CO115 transfected with full-length CEA-cDNA; CO115–2C2: CO115 transfected with truncated CEA-cDNA. (**B**) Flow cytometry analysis of CEA expression by the three colon carcinoma cell lines used for the metastatic potential assay: LS174T, LoVo and CO112. The same specific mAb for the CEA Gold 2 epitope (grey shading) and an irrelevant IgG (no shading) were used. CEA expression was checked by FACS before any in vivo experiment using these cell lines

### CEA measurements

The concentration of CEA in the culture supernatant and in the serum of nude mice bearing different subcutaneous (s.c.) tumours was determined using a radioimmunoassay (ELSA2-CEA in vitro test®, CIS Bio International, Gif-Sur-Yvette, France).

### Animal subcutaneous tumour inoculation and tumour resection

Experiments were performed on 6- to 8-week-old female BALB/c nude mice (Iffa-Credo, L'Arbresle, France). Mice had free access to feed and water and were housed five mice per cage under specific pathogen-free conditions. All experimental protocols were performed in accordance with the French guidelines for experimental animal studies. CO115 and derived tumours were propagated by serial s.c. transplantation into the right flanks of the mice. Tumour size was evaluated by measuring the tumour diameters (three dimensions) and the volume was calculated by the formula: vol = $r_1 \times r_2 \times r_3 \times 4\pi/3$ where r is the radius.

For s.c. tumour resection, the mice were anaesthetized with 2,2,2 tribromoethanol 0.5 mM in 10% ethanol and 90% sodium chloride (10 ml $kg^{-1}$). During tumour resection, special care was taken to avoid the release of tumour cells into the tumour venous effluent by clamping blood vessels.

### Metastatic potential assay

The potential of cell lines to form experimental metastases was examined in BALB/c nude mice after intrasplenic (i.s.) injection according to Kozlowski et al (1984). Special care was taken to obtain single cell preparations to eliminate the possibility of liver metastases due to vascular embolism. Cells were incubated for 15 min at 37°C with trypsin (2.5 mg $ml^{-1}$) and then washed once with RPMI-1640 10% FCS and twice with PBS without $Ca^{++}$ or $Mg^{++}$. Cells were kept for 30 min at 4°C in PBS before the last washing. Mice were anaesthetized and, after a laparotomy, given an i.s. injection of $5 \times 10^5$ cells in 50 µl through a 26-gauge needle. All groups were sacrificed when mice in any group became moribond. Autopsies were performed and the presence of metastases was determined by macroscopic inspection and confirmed histologically. Tumour burden was assessed according to the classification of Giavazzi et al (1986): 0, no tumour deposit, i.e. tumour-free; I, histological evidence of tumour growth or one isolated metastasis; II, fewer than ten tumour foci, < 1–2 mm in diameter; III, 10–100 tumour foci, 3–5 mm in diameter; IV, more than 100 tumour foci, > 5 mm in diameter.

### Statistical analysis

The Kruskal–Wallis test or the $\chi^2$ distribution was performed to analyse the statistical significance of the effect of the circulating

© 1999 Cancer Research Campaign

CEA levels, the type of cells injected and the s.c. tumour resection status on the metastatic potential assay.

### RESULTS

### Transfection of full-length CEA-cDNA

The full-length CEA-cDNA construct was transfected into a CEA-negative subclone from the human colon carcinoma cell line CO115 by the calcium phosphate precipitation method. The transfected cells were selected first with the neomycin analogue G418. In the G418-resistant cells, 10% were found to be CEA-positive by flow cytometry using a pool of CEA-specific mAb labelled with fluoroscein. The cells were sorted and cloned by limiting dilution. Out of 960 initials wells, 100 clones were isolated. Eight of them were further characterized, but only one (CO115-5F12) was used for the following experiments.

### Expression of CEA in CO115 cells transfected with full-length or truncated CEA-cDNA

FACS analysis showed that the parental cell line CO115 expressed none of the four CEA-specific epitopes (Gold 1–4) but only the Gold 5 epitope which is shared with the non-specific cross-reacting antigen NCA (Hammarstrom et al, 1989). After transfection with the full-length CEA-cDNA, the clones were found to express all the CEA epitopes Gold 1–5. The CEA molecule expressed by the transfected clones was PI-PLC sensitive confirming a GPI anchor of the molecule. We selected clone CO115-5F12 for the following study because it was one of the higher CEA expressers (Figure 1A). After transfection with truncated CEA-cDNA, no membrane expression was detectable in any of the transfected clones (Figure 1A). This was illustrated with clone CO115-2C2 which was selected for the present study because it has been shown to secrete up to 13 μg CEA per $10^6$ cells within 72 h in the culture supernatant (Terskikh et al, 1993).

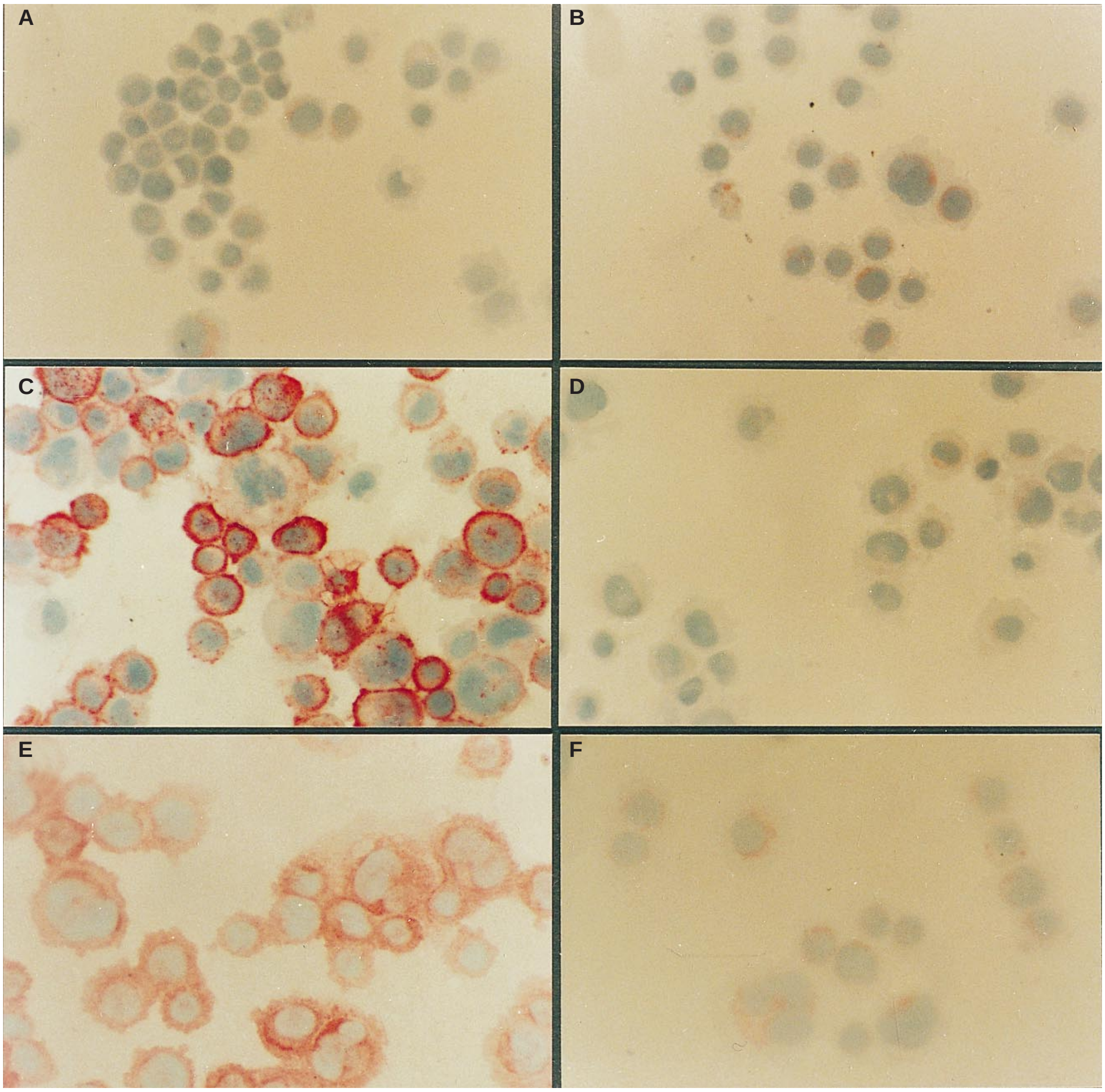

**Figure 2** Immunocytochemical analysis of CEA expression of full-length and truncated CEA-cDNA-transfected clones and parental cell line CO115. The parental cell line, CO115, did not express CEA (**A**). The full-length CEA-cDNA-transfected clone, CO115-5F12, expressed CEA mainly at the membrane level (**C**); whereas in the truncated CEA-cDNA transfected clone, CO115-2C2, the CEA expression was limited to the cytoplasm (**E**). An irrelevant IgG was used as negative control with the three cell lines (**B**, CO115; **D**, CO115–5F12; **F**, CO115-2C2)

 © 1999 Cancer Research Campaign

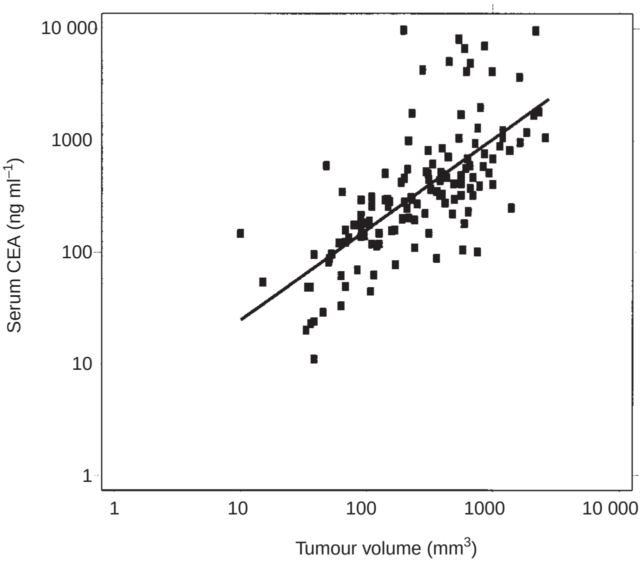

**Figure 3** Serum CEA concentration expressed as a function of tumour volume in CO115-2C2 tumour-bearing mice. The regression equation was given by $\log_{10}$ (serum CEA) = 0.574 + 0.814 $\log_{10}$ (tumour volume). The linear correlation coefficient of this equation is $r = 0.704$. No circulating CEA was detectable in mice bearing the CO115 or the CO115-5F12 tumours

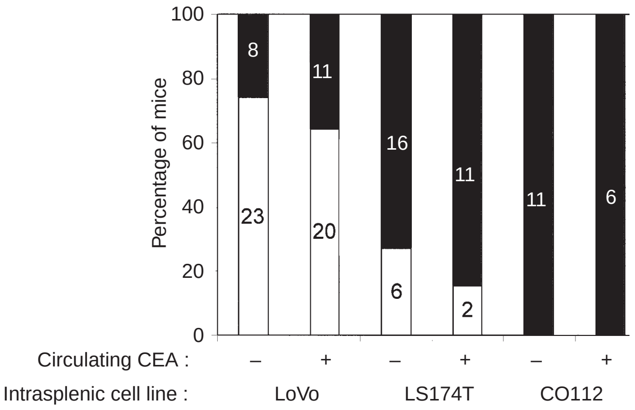

**Figure 4** Summary of the metastatic potential assay. The mice described in Table 1 were divided into two groups: mice with no circulating CEA (i.e. bearing s.c. the CO115 or CO115-5F12 tumour, or no s.c. tumour) and mice with circulating CEA (i.e. bearing the s.c. CO115-2C2 tumour). The results are expressed as a percentage of mice without □ or with liver metastasis ■ whatever the grade following i.s. injection of the different cell lines, i.e. LoVo, LS174T or CO112. The exact number of mice in each group is indicated

CEA expression following the two different transfections was also analysed by immunocytochemistry (Figure 2). Binding of the anti-CEA antibodies revealed a clear membrane expression of the antigen in clone CO115-5F12, whereas only cytoplasmic expression was found in clone CO115-2C2. This observation confirmed the results of our previous study where we demonstrated that deletion of the 78 bp at the 3′ end of the CEA-cDNA, encoding the 26-amino acid hydrophobic domain, induced active secretion of CEA (Terskikh et al, 1993).

### Development of an animal model with different levels of circulating CEA: CO115-2C2 tumour-bearing nude mice

The parental CO115 cells and the transfected clones, CO115-5F12 and CO115-2C2, were grafted s.c. into the right flanks of BALB/c nude mice and the resulting tumours were propagated by serial s.c. transplantation. Serum-circulating CEA was measured at different times and expressed as a function of the tumour volume. Mice bearing the CO115-2C2 tumours showed a clear correlation between these two parameters (Figure 3). The regression equation was given by $\log_{10}$ (serum CEA) = 0.574 + 0.814 $\log_{10}$ (tumour volume). The linear correlation coefficient of this equation is $r = 0.704$. For tumours ranging from 100 to 1000 mm$^3$, we observed serum CEA values ranging from 100 to 1000 ng ml$^{-1}$. No CEA was detectable in the serum of mice bearing the CO115 or the CO115-5F12 tumours.

### Metastatic potential assay

Three human colon carcinoma cell lines were selected for the metastatic potential assay: LS174T (ATCC CL188), LoVo (ATCC CCL229) and CO112 (Mach et al, 1974) according to their high CEA expression which was confirmed by FACS analysis before any in vivo experimentation (Figure 1B).

For the metastatic potential assay, mice were first s.c. grafted with the CO115, CO115-5F12 or CO115-2C2 tumour. When the tumours had reached a volume of about 100–200 mm$^3$, blood samples were taken and CEA was measured in the serum. Depending on the tumour volume, mice bearing CO115-2C2 tumours showed circulating CEA levels ranging from 22 to 818 ng ml$^{-1}$ (Table 1). Intrasplenic injections of LS174T, LoVo or CO112 cells were performed the next day. Special care was taken to inject single cells because cell aggregates could be arrested in capillary beds enhancing the experimental metastatic potential of the cells (Liotta et al, 1976).

With the LoVo and LS174T cell lines, two different protocols were performed. In the first procedure, the s.c. tumours were kept on the mice after the i.s. injection of the LS174T or the LoVo cells. Because of the cumulated morbidity due to the presence of both the s.c. and the i.s. tumours, this procedure led to the necessity to sacrifice the mice at a relatively short time after the i.s. injection, that is day 11 and 29 post i.s. injection for the LS174T and LoVo cells respectively. To avoid this problem, a second protocol was designed in which the s.c. tumours were resected on day 7 after the i.s. injection of LS174T or LoVo cells. This allowed us to keep the mice alive for up to 27 and 42 days post i.s. injection of the LS174T and LoVo cells respectively. Only the second protocol was used for the CO112 cell line. A kinetic analysis of circulating CEA showed a clear increase for the 7 days between the i.s. injection of cells (D0) and s.c. tumour resection (D7) (Table 1). The CEA depletion occurred in about 2 days giving, for example, values of 86.5, 41.5 and 39.5 ng ml$^{-1}$ 24 h after s.c. tumour resection in mice with 960, 327 and 283 ng CEA per ml, respectively, the day before resection.

The experimental liver metastases data are presented in Table 1. Whatever the s.c. tumour and the resection status, i.s. injection of LoVo cells induced metastases which did not exceed grade II. Grades III and IV were observed more frequently with LS174T cells and, in particular, with CO112 cells. This difference between the ability of the three cell lines to induce liver metastases was shown to be statistically significant ($P = 0.0001$ by the Kruskal–Wallis test). The s.c. tumour resection affected liver metastases development essentially for LS174T cells for which

© 1999 Cancer Research Campaign 

grade III and IV were more frequent after resection as compared with mainly grade II without resection. A global analysis of the resection status of the LS174T and LoVo cells showed a difference close to statistical signification ($P = 0.0512$). Whatever the protocol used (cell line, resection status), circulating CEA levels did not affect the liver metastases development ($P = 0.734$). In all cases, the CO115-2C2 tumour-bearing mice developed liver metastases close to those observed in non-CEA secreting tumour-bearing mice. All these data are summarized in Figure 4 where the results are expressed in percentage of mice with or without liver metastases as a function of the presence or absence of circulating CEA and the type of i.s. injected cell line. In this summarized form, there appears to be more metastases in the mice with circulating CEA but the difference remains non-significant: $P = 0.29$ and $P = 0.36$ for the LoVo and LS174T cell lines respectively ($\chi^2$ distribution).

### DISCUSSION

Circulating CEA is related to poor prognosis in colorectal cancer patients. Because the precise involvement of this tumour marker in the development of liver metastases cannot be analysed in humans, many studies have been conducted using animal models to try to demonstrate a connection between cause and effect. Most of the studies designed to address this question were based on the hypothesis that the adhesive properties of CEA might facilitate the development of liver metastases from colorectal cancer (Hostetter et al, 1990; Jessup et al, 1993*a*; Tibbetts et al, 1993; Thomas et al, 1995; Landuzzi et al, 1996). Recently, some authors suggested that CEA could enhance liver metastases via induction of cytokines in Kupffer cells (Gangopadhyay et al, 1996; Edmiston et al, 1997).

The adhesive properties of CEA have been demonstrated in vitro with different colon carcinoma cell lines expressing CEA and NCA or CHO cells transfected with CEA or NCA cDNA. Such cells are able to form homotypic aggregates (CEA–CEA or NCA–NCA) and to a lower extent heterotypic ones (CEA–NCA) (Benchimol et al, 1989; Oikawa et al, 1989; Zhou et al, 1990). Two different CEA epitopes are presumed to be necessary for CEA–CEA interactions (Jessup et al, 1993*b*). Zhou et al (1993) proposed an interaction model involving the N-terminal and A3 domains. Contribution of the N-terminal domain was confirmed by different authors but that of the A3 domain was contested (Jessup et al, 1993*b*; Hashino et al, 1994).

Whatever the domains involved, CEA could promote the development of liver metastases through homotypic or heterotypic adhesion between carcinomas cells themselves or between carcinoma cells and CEA trapped at the surface of Kupffer cells (Toth et al, 1992; Jessup et al, 1993*a*). Using ten human colon carcinoma cell lines, Tibbetts et al (1993) demonstrated that there is a connection between membrane CEA expression and the development of experimental liver metastases following i.s. injection of the cells. The high CEA-expressing cell lines gave metastases more frequently than the low-expressing ones, but there was not an absolute correlation. Hashino et al (1994) used CHO cells transfected with the CEA-cDNA and obtained 100% metastases with the CHO cells expressing CEA as compared with no metastasis with the CHO mock-transfected cells. In all these studies, the development of liver metastases was correlated with the early retention of the cells in the liver and the in vitro adhesion properties of the cells (Jessup et al, 1993*a*). The relationship between adhesion properties and the development of liver metastases was confirmed in a study by Landuzzi et al (1996) who reported an inhibition of lung metastases after transfection of the CEA-cDNA in the human rhabdomyosarcoma cell line RD/18. The decrease of lung metastases was clearly related to a decrease of adhesion properties of the cells after transfection. In a homotypic adhesion test, the transfected cells gave more than 60% single cells as compared with about 20% for the parental cell line. On the basis of these studies on membrane CEA and liver metastases, it appears that CEA is certainly one of the molecules involved in cell–cell interaction, but many other molecules probably also participate in this phenomenon. The nature and the number of these molecules can vary from one type of cell to another, accounting for conflicting results reported by different authors (Jessup et al, 1993*a*; Landuzzi et al, 1996). In all the cases, the single cell nature of the cell preparation used for the induction of experimental liver metastases is a primordial parameter. Cell aggregates can arrest in capillary beds enhancing the experimental metastatic potential of cells (Liotta et al, 1976).

To analyse the involvement of circulating CEA on the development of liver metastases from colorectal carcinoma in nude mice, Jessup et al (1993*a*) and Hostetter et al (1990) injected CEA i.v. 30 min before i.s. injection of different human colon carcinoma cell lines. A 40-μg CEA dose enhanced experimental liver metastases following injection of three poorly metastatic cell lines (KM-12c, MIP-101 and Clone A) or highly metastatic ones (CCL 235, HT 29 and mHC 1410). The authors observed a significant correlation between the formation of experimental metastases and the early retention of tumour cells in the liver. From our point of view, the limitation of these studies was the use of an i.v. bolus injection of CEA to obtain circulating CEA in the nude mouse model. The 40-μg dose of injected CEA gave about 400 ng CEA per ml serum 4 h post-injection, but only 12 ng CEA per ml serum 20 h later.

Although according to the authors, this experimental procedure gave about 75 ng CEA per mg liver protein 4 h after CEA injection, this appears to us to be a situation quite different from that observed in patients with increasing circulating CEA levels. In such cases, the CEA from the tumour is shed into the circulation and cleared by the liver where it binds first to Kupffer cells in which the terminal sialic acid residues are removed. Asialo-CEA is then transferred to hepatocytes where it is degraded (Hostetter et al, 1990; Gangopadhyay et al, 1996). An increase in circulating CEA levels thus corresponds to the saturation of the liver sites by the CEA shed from the tumour.

For this reason, we developed a new animal model mimicking the human situation of increasing circulating CEA levels. Because human colon carcinoma s.c. grafted into nude mice give no detectable circulating CEA (Folli et al, 1993), we used a CEA-negative clone of the colon carcinoma cell line CO115 transfected with truncated CEA-cDNA lacking the 78 base pairs at the 3′ region encoding the 26-amino acid hydrophobic domain. This transfected cell line, CO115-2C2, was shown to actively secrete CEA into the culture (Terskikh et al, 1993). When grafted s.c. into nude mice, CO115-2C2 cells gave CEA-secreting tumours producing circulating CEA levels ranging from 100 to 1000 ng $ml^{-1}$ with s.c. tumours as small as 100–1000 $mm^3$. We observed a clear correlation between circulating CEA levels and tumour volume (Figure 3).

We used this animal model to analyse the involvement of circulating CEA in the development of liver metastases induced experimentally by i.s. injection of human colon carcinoma cells (Kozlowski et al, 1984). We focused our attention on three cell lines (LS174T, LoVo and CO112) which express high amounts of membrane CEA as determined by FACS analysis. Depending on the i.s. injected cell line and the protocol used (involving or not s.c. tumour resection), we obtained all the possible grades of liver

*© 1999 Cancer Research Campaign*

metastases (Table 1). The s.c. tumour resection allowed us to keep the animals alive longer: 42 and 27 days as compared with 29 and 11 days without resection for LoVo and LS174T respectively. Circulating CEA did not significantly affect the number or the size of the liver metastases with either of the cell lines used or protocols tested (Table 1 and Figure 4).

Our experimental model, which closely resembles the clinical situation, did not allow us to demonstrate any effect of circulating CEA on the development of liver metastases. This animal model in which there is a direct relationship between the volume of the tumour and the level of circulating CEA could be used to study, for example, the influence of circulating CEA on tumour targeting with anti-CEA mAbs (Buchegger et al, 1995).

The purpose of the present study was not to analyse the effect of membrane CEA on the development of liver metastases from colorectal cancer since this parameter is specific for each tumour and cannot be modified by any treatment. Our purpose was to evaluate the eventual effect of circulating CEA on the development of liver metastases. The question was addressed by using three cell lines expressing high levels of membrane CEA. On the basis of the results obtained with these three cell lines, we conclude that circulating CEA is not involved in the development of liver metastases from colorectal cancers. Membrane CEA could enhance liver metastases by the formation of cell aggregates trapped in the small venules of the liver. In this case, CEA would be responsible for the attachment of carcinoma cells in clusters, but the binding of these clusters in the liver is only a mechanistic phenomenon and is not due to homophilic interactions between circulating cancer cells and CEA expressed by Kupffer cells.

### ACKNOWLEDGEMENTS

The authors thank S Bouquié, R Dietz, G Heintz for excellent technical assistance, M Brissac for performing the animal experiments, M Radal for the immunocytochemistry experiments, L Désiré for the statistical analysis, SL Salhi for editorial assistance, CisBio International for providing the ELSA2-CEA in vitro test and the radioanalysis laboratory at the CRLC for performing the CEA assays. This work was supported by the Ligue Nationale Contre le Cancer (Comités de l'Hérault et de l'Aude) the Fondation Gustave Prévot and Région Languedoc-Roussillon.

### REFERENCES

Benchimol S, Fuks A, Jothy S, Beauchemin N, Shirota K, Stanners CP (1989) Carcinoembryonic antigen, a human tumor marker, functions as an intercellular adhesion molecule. *Cell* **57**: 327–334

Buchegger F, Mach JP, Pèlegrin A, Gillet M, Vogel CA, Buclin T, Ryser JE, Delaloye B and Bischof-Delaloye A (1995) Radiolabeled chimeric anti-CEA monoclonal antibody compared with the original mouse monoclonal antibody for surgically treated colorectal carcinoma. *J Nucl Med* **36**: 420–429

Carrel S, Sordat B and Merenda C (1976) Establishment of a cell line (CO115) from a human colon carcinoma transplanted into nude mice. *Cancer Res* **36**: 3978–3984

Edmiston KH, Gangopadhyay A, Shoji Y, Nachman AP, Thomas P and Jessup JM (1997) In vivo induction of murine cytokine production by carcinoembryonic antigen. *Cancer Res* **57**: 4432–4436

Folli S, Pèlegrin A, Chalandon Y, Yo X, Buchegger F, Lejeune F and Mach J-P (1993) Tumor Necrosis Factor can enhance radio-antibody uptake in human colon carcinoma xenografts by selective increase of vascular permeability. *Int J Cancer* **53**: 829–836

Gangopadhyay A, Bajenova O, Kelly TM and Thomas P (1996) Carcinoembryonic antigen induces cytokine expression in kupffer cells: implications for hepatic metastasis from colorectal cancer. *Cancer Res* **56**: 4805–4810

Giavazzi R, Campbell DE, Jessup JM, Cleary K and Fidler IJ (1986) Metastatic behaviour of tumor cells isolated from primary and metastatic human colorectal carcinomas implanted into different sites in nude mice. *Cancer Res* **46**: 1928–1933

Hammarstrom S, Shively JE, Paxton RJ, Beatty BG, Larson A, Ghosh R, Bormer O, Buchegger F, Mach J-P, Burtin P, Seguin P, Darbouret B, Degorce F, Sertour J, Jolu J-P, Fuks A, Kalthoff H, Schmiegel W, Arndt R, Kloppel G, von Kleist S, Grunert F, Schwarz K, Matsuoka Y, Kuroki M, Wagener C, Weber T, Yachi A, Imai K, Hishikawa N and Tsujisaki M (1989) Antigenic sites in carcinoembryonic antigen. *Cancer Res* **49**: 4852–4858

Hardman N, Lee Gill L, de Winter RFJ, Wagner K, Hollis M, Businger F, Ammaturo D, Buchegger F, Mach J-P and Heusser C (1989) Generation of a recombinant human-mouse chimaeric monoclonal antibody directed against human carcinoembryonic antigen. *Int J Cancer* **44**: 424–433

Hashino J, Fukuda Y, Oikawa S, Nakazato H and Nakanishi T (1994) Metastatic potential in nude mice of Chinese hamster ovary cells expressing human carcinoembryonic antigen. *Biochem Biophys Res Commun* **200**: 1748–1753

Hostetter RB, Augustus LB, Mankarious R, Chi KF, Fan D, Toth C, Thomas P and Jessup JM (1990) Carcinoembryonic antigen as a selective enhancer of colorectal cancer metastasis. *J Natl Cancer Inst* **82**: 380–385

Jessup JM, Petrick AT, Toth CA, Ford R, Meterissian S, O'Hara CJ, Steele G Jr, and Thomas P (1993*a*) Carcinoembryonic antigen: enhancement of liver colonisation through retention of human colorectal carcinoma cells. *Br J Cancer* **67**: 464–470

Jessup JM, Kim JC, Thomas P, Ishii S, Ford R, Shively JE, Durbin H, Stanners CP, Fuks A, Zhou H, Hansen HJ, Goldenberg DM and Steele G Jr (1993*b*) Adhesion to carcinoembryonic antigen by human colorectal carcinoma cells involves at least two epitopes. *Int J Cancer* **55**: 262–268

Kozlowski JM, Fidler IJ, Campbell D, Xu Z, Kaighn ME and Hart IR (1984) Metastatic behavior of human tumor cell lines grown in the nude mouse. *Cancer Res* **44**: 3522–3529

Landuzzi L, Frabetti F, Rossi I, Griffoni C, De Giovanni C, Nicoletti G, Nanni P, Miniero R, Palmieri G, Santoni A and Lollini PL (1996) Expression of transduced carcinoembryonic antigen gene in human rhabdomyosarcoma inhibits metastasis. *Cancer Res* **56**: 4503–4508

Liotta LA, Saidel MG and Kleinerman J (1976) The significance of hematogenous tumor cell clumps in the metastatic process. *Cancer Res* **36**: 889–894

Lucha PA Jr, Rosen L, Olenwine JA, Reed JF 3rd, Riether RD, Stasik JJ Jr and Khubchandani IT (1997) Value of carcinoembryonic antigen monitoring in curative surgery for recurrent colorectal carcinoma. *Dis Colon Rectum* **40**: 145–149

Mach J-P, Carrel S, Merenda C, Sordat B and Cerottini J-C (1974) In vivo localisation of radiolabelled antibodies to carcinoembryonic antigen in human colon carcinoma grafted into nude mice. *Nature* **248**: 704–706

Oikawa S, Inuzuka C, Kuroki M, Matsuoka Y, Kosaki G and Nakazato H (1989) Cell adhesion activity of non-specific cross-reacting antigen (NCA) and carcinoembryonic antigen (CEA) expressed on cho cell surface: homophilic and heterophilic adhesion. *Biochem Biophys Res Commun* **164**: 39–45

Pèlegrin A, Terskikh A, Hayoz D, Chalandon Y, Olsson NO, Folli S, Buchegger F, Kromer B, Schwarz K, Martin M, Martin F and Mach J-P (1992) Human carcinoembryonic antigen cDNA expressed in rat carcinoma cells can function as target antigen for tumor localization of antibodies in nude rats and as rejection antigen in syngeneic rats. *Int J Cancer* **52**: 110–119

Pignatelli M, Durbin H and Bodmer WF (1990) Carcinoembryonic antigen functions as an accessory adhesion molecule mediating colon epithelial cell-collagen interactions. *Proc Natl Acad Sci USA* **87**: 1541–1545

Terskikh A, Mach JP and Pèlegrin A (1993) Marked increase in the secretion of a fully antigenic recombinant carcinoembryonic antigen obtained by deletion of its hydrophobic tail. *Mol Immunol* **30**: 921–927

Thomas P, Gangopadhyay A, Steele G Jr, Andrews C, Nakazato H, Oikawa S and Jessup JM (1995) The effect of transfection of the CEA gene on the metastatic behavior of the human colorectal cancer cell line MIP-101. *Cancer Lett* **92**: 59–66

Tibbetts LM, Doremus CM, Tzanakakis GN and Vezeridis MP (1993) Liver metastases with 10 human colon carcinoma cell lines in nude mice and association with carcinoembryonic antigen production. *Cancer* **71**: 315–321

Toth CA and Thomas P (1992) Liver endocytosis and Kupffer cells. *Hepatology* **16**: 255–266

Zhou H, Fuks A and Stanners CP (1990) Specificity of intercellular adhesion mediated by various members of the immunoglobulin supergene family. *Cell Growth Differ* **1**: 209–215

Zhou H, Fuks A, Alcaraz G, Bolling TJ, Stanners CP (1993) Homophilic adhesion between Ig superfamily carcinoembryonic antigen molecules involves double reciprocal bonds. *J Cell Biol* **122**: 951–960

*© 1999 Cancer Research Campaign*
